# Supplementary material for: Effectiveness of antifungal treatments during chytridiomycosis epizootics in populations of an endangered frog
Source: PeerJ. 2022 Jan 5;10:e12712. doi: 10.7717/peerj.12712 (PMC8742549; doi:10.7717/peerj.12712)
Supplement: Supplemental Information 1 [file peerj-10-12712-s001.pdf]

Supporting Information

Effectiveness of antifungal treatments during chytridiomycosis epizootics in populations of an endangered frog

Roland A. Knapp Maxwell B. Joseph Thomas C. Smith
Ericka E. Hegeman Vance T. Vredenburg James E. Erdman, Jr.
Daniel M. Boiano Andrea J. Jani Cheryl J. Briggs

Author Contributions

Roland Knapp obtained the permits for all treatments, designed and implemented all of the treatments except at Treasure, conducted post-treatment surveys, managed the datasets, conducted final analyses of all of the treatment datasets except the capture-mark-recapture (CMR) dataset from the LeConte and Treasure treatments, and wrote most of the manuscript.

Maxwell Joseph assisted with the Barrett-2009 and Dusy-2010 treatments and associated post-treatment surveys, analyzed the CMR dataset from the LeConte experiment and wrote the associated text, provided input on most of the analyses, and reviewed all drafts of the manuscript.

Thomas Smith assisted with the LeConte and Treasure treatments, conducted post-treatment surveys for multiple treatments, analyzed the results of the Treasure treatment and wrote some of the associated text, conducted preliminary analyses of

the Barrett-2009, Dusy-2010, and Dusy-2012 treatments, and reviewed all drafts of the manuscript.

Ericka Hegeman assisted with the Treasure treatment, conducted post-treatment surveys for multiple treatments, managed the datasets, conducted preliminary analyses of the Barrett-2009 and Dusy-2010 treatments and wrote some of the associated text, and reviewed all drafts of the manuscript.

Vance Vredenburg assisted with the design and implementation of the Dusy-2012 treatment, including supporting the development of the *J. lividum* qPCR assay, culturing of the *J. lividum* used in the treatment, and qPCR analysis of *J. lividum* samples.

James Erdman conducted pre- and post-treatment surveys at Treasure, implemented the Treasure treatment, and reviewed all drafts of the manuscript.

Daniel Boiano assisted with the LeConte treatment, facilitated the National Park Service permitting of all treatments conducted in Sequoia and Kings Canyon National Parks, and reviewed all drafts of the manuscript.

Andrea Jani assisted with the LeConte treatment, and conducted the Dusy Basin zoospore pool study, provided the associated dataset, conducted preliminary analyses, and wrote some of the associated text, and reviewed all drafts of the manuscript.

Cheryl Briggs provided modeling results that motivated the treatments, assisted with the design of treatments, and reviewed all drafts of the manuscript.

General Methods

Visual encounter surveys

We counted *R. sierrae* of all life stages (adults: $\geq$ 40 mm snout-vent length (SVL); subadults: $<$ 40 mm; tadpoles) using diurnal visual encounter surveys (VES) of the entire

water body shoreline and the first 100 m of inlet and outlet streams. VES is commonly used in studies of MYL frogs to estimate the abundance of all life stages present at a site (e.g., Knapp & Matthews, 2000; Vredenburg et al., 2010; Knapp et al., 2016). Counts are highly repeatable (Knapp & Matthews, 2000), but underestimate the number of animals present.

Itraconazole treatment

For all itraconazole treatments, we held animals assigned to the "treated" group in large mesh pens (2 m x 2 m x 0.75 m) for the duration of the multi-day treatment period (Figure S1). Holding animals in pens assured that each animal could be captured and treated on each day of the treatment period; this would not have been possible if animals needed to be recaptured from the ponds each day. Pens were anchored in the littoral zone of the study lakes, and contained shallow water and shoreline habitats for basking as well as deeper water habitat (up to 0.7 m). Animals assigned to the untreated "control" group were held in pens only 3-24 hr and then released back into the lake; temporarily holding these individuals, rather than releasing them immediately, guaranteed they were not recaptured and resampled.

Although it would have been ideal to hold animals from both the treated and control groups in pens for the duration of the capture and treatment periods, doing so could have produced spurious and misleading results. Bd transmission is expected to increase with frog density (Rachowicz & Briggs, 2007), and holding untreated control animals in pens at relatively high density could therefore have increased their Bd loads more than would be expected for animals in the treated group that were given daily antifungal baths. This would have biased the outcome toward lower post-treatment survival of control animals compared to treated animals even if the antifungal treatment itself had no effect on post-treatment survival. Assuming that holding animals in pens for several days has some

negative effect (due to increased Bd transmission even in treated frogs, and lack of feeding opportunities), if our study design caused biases they should be conservative, i.e., reducing the survival of treated animals relative to control animals. Including a second control group, in which untreated animals were held for the entire treatment period, could have been informative, but would have substantially reduced the number of animals included in the existing treated and control groups and potentially affected our ability to detect treatment effects (due to reduced statistical power).

To conduct the antifungal treatments, on each day during the multi-day treatment period we transferred all animals in the treated group from pens to small plastic tubs that contained a dilute solution (1.5 mg L^{-1}; Garner et al., 2009) of the antifungal drug itraconazole (trade name = Sporonox). We treated frogs in batches of approximately 50, and tadpoles in batches of approximately 100. The volume of itraconazole solution varied between 2 and 5 L, and was varied between batches based on the number and life stage of animals being treated to allow all individuals to submerge fully. After 10 minutes of itraconazole exposure, animals were transferred from the tubs back to the pens. After the final treatment, we released all animals from the pens back into the study lakes. To determine treatment effectiveness, we swabbed all animals or a subset (depending on the experiment) following their initial capture and at the end of the treatment period.

Janthinobacterium lividum qPCR assay

The *J. lividum* used to develop the real-time PCR assay was a strain provided by Reid Harris, Department of Biology, James Madison University. *J. lividum* DNA was extracted from a pure culture of this strain using a Qiagen DNeasy blood and tissue kit following the manufacturer's protocol. The DNA collected was amplified using methods in Harris et al. (2009). The PCR product was viewed on a 1% agarose gel, and the 500 bp product was sequenced.

Primers were designed to be specific to *J. lividum* and sequences were:

- Jliv_For3 ATGCCACCGACGGCTACCA
- Jliv_Rev1 ACGGCGGGATGGTCATCAC

The minor groove binder probe sequence was:

- JLIVT 6FAM AACATCGTTTGCTGTCCGTTGA MGBNFQ

After assay optimization, the 25 μL reaction volumes included 0.5 μL of each primer at a concentration of 10 μM, 0.375 μL of MGB probe at a concentration of 250 nM, 12.5 μL of Taqman MasterMix (Applied Biosystems), 1.25 μL BSA, and 5 μL of template. The amplification conditions consisted of an initial cycle of 2 min at 50°C and 10 min at 95°C, followed by 50 cycles at 95°C for 15 s, 58°C for 30 s, and 65°C for 45 s. To create standards, DNA was extracted from pure cultures of *J. lividum* with an UltraClean microbial DNA isolation kit (MoBio), and diluted to 10^4, 10^3, 10^2, and 10^1 *J. lividum* genome equivalents. A standard curve was generated for each 96-well plate to estimate the number of *J. lividum* genome equivalents in sample extracts.

Statistical analyses

Simple and multilevel models using the brms package

When using the brms package, we started with models that included all relevant population-level ("fixed") effects and their interactions ("full model"), then checked model fit using visualizations of leave-one-out ("LOO") probability integral transformations (Gelman et al., 2013; Vehtari, Gelman & Gabry, 2017). When data contained possible hierarchical structure (which could result in non-independence), we included group-level ("random") effects in the full model. Bd load and frog counts were the most commonly

modeled response variables, and models that used a negative binomial family generally produced the best fit. However, when appropriate, we also evaluated the fit of models that used other model families, including Poisson and zero-inflated negative binomial. We compared fits of models using LOO cross-validation and the *loo* package (Vehtari, Gelman & Gabry, 2017). For all models, we used brms defaults for priors, number of chains (4), and warmup and post-warmup iterations (1000 for each). We evaluated the adequacy of posterior samples using trace plots, Gelman-Rubin statistics (Rhat), and measures of effective sample size ("bulk-ESS", "tail-ESS"). When using negative binomial models (most analyses), the Bd load data were rounded to integer values to produce count data.

When necessary, we developed distributional models in which predictor terms are specified for other parameters of the response distribution instead of only the mean (e.g., negative binomial overdispersion ("shape"), zero-inflation ("zi"); see brms vignette, "Estimating distributional models with brms": https://paul-buerkner.github.io/brms/articles/brms_distreg.html). The overdispersion parameter ϕ controls the variance of the negative binomial distribution relative to the expected value μ, such that the variance of the negative binomial distribution is $\mu + \mu^2/\phi$. Modeling effects on overdispersion and zero-inflation can be important for improving model fit. For example, itraconazole treatment can reduce not only mean Bd load, but also the variation around the mean (i.e, overdispersion) and amount of zero-inflation. Improving model fit was our primary interest in using distributional models, and not gaining insights into the causes of overdispersion or zero-inflation. Therefore, when we used distributional models, we limit our descriptions of model results largely to effects of predictors on the mean.

Treatment-specific Methods and Results

Estimating the zoospore pool from water samples – Dusy Basin

Methods

As part of the Dusy Basin itraconazole treatment experiment (Table S1), we sampled the zoospore pool in each of the five study ponds before and after the treatments (July 23-25 and August 21-24, respectively). Water samples (six per pond) were collected by filtering pond water through a 0.22 mm pore polyethersulfone filter (Sterivex-GP; Millipore), until the filter clogged. Filters were immediately amended with sucrose lysis buffer (40 mmol/l 1 EDTA, 50 mmol/l 1 Tris-HCl, 750 mmol/l sucrose, pH adjusted to 8.0). We extracted DNA using the DNEasy Blood and Tissue kit (Qiagen). Bd concentrations in water samples (environmental "Bd loads") were quantified using qPCR (see Jani, Knapp & Briggs, 2017 for details), and Bd load was normalized to a 1-liter sample volume. For each sample, we ran three technical replicates, each from an independent sample dilution and on an independent PCR run. To minimize PCR inhibition, we diluted DNA extracts 50-fold based on pilot tests using undiluted, ten-, fifty-, and one hundred-fold dilutions. Finally, we included 21 negative controls: nine no-template-controls (three per PCR plate) and 12 field-collected negative controls (six water samples collected from a pond where all frogs were Bd-negative, each with two technical replicates).

The 21 negative controls yielded no false-positive PCR reactions. However, it is common for technical replicates of environmental DNA samples to have a high rate of false-negatives due to low quantities of target DNA or PCR inhibitors in samples (Mosher, Huyvaert & Bailey, 2018), and this was the case in our study. Despite the fact that all five study ponds contained relatively large numbers of early life stage *R. sierrae* characterized by high Bd loads, of the 180 total replicates (5 ponds x 6 water samples x 3 technical replicates x 2 time periods (before and after treatment)), 49% had a Bd load

= 0. Because the five ponds in the experiment were clearly Bd-positive, we considered replicates with Bd load = 0 to be false negative replicates, which we excluded from the analysis of the effect of itraconazole treatment on the Bd zoospore pool. This resulted in one pond assigned to the treated group being dropped from the analysis due to a lack of any Bd-positive replicates. The effect of itraconazole treatment on Bd concentrations on filters was evaluated using the model *bd_load ~ (pre_post x treatment) + (1 | sample_id)* (family = negative binomial, pre_post = [before treatment, after treatment], treatment = [treated, control], sample_id included as a group-level effect to account for technical replicates).

Results

Prior to the itraconazole treatment, zoospore pools (measured as Bd load on collected filters) in the ponds assigned to the control and treated groups were similar (Figure S2). After treatment, zoospore pools in control ponds may have increased slightly, but remained relatively constant in treated ponds (Figure S2). Model results indicated that the estimated effects of treatment, basin, and the (treatment x basin) interaction term were all unimportant (Table S5). Therefore, assuming that the sampling method was adequate to accurately quantify pond-wide zoospore concentrations, the treatment of even a relatively large fraction of the resident *R. sierrae* in the study ponds did not measurably alter the zoospore pools. To avoid the high number of false-negative filters obtained using our methods, future studies attempting to quantify zoospore pools should consider using methods that allow filtering of larger volumes of water.

Itraconazole treatment of adults – LeConte Basin

Methods – Hidden Markov model

We tracked the fates of individual animals in the LeConte population using a multi-state hidden Markov model. We consider three possible observations for each individual $i = 1, ..., M$ in primary period $t = 1, ..., T$, on secondary period $j = 1, ..., J_t$, where T is the total number of primary periods and J_t is the number of secondary periods in primary period t:

- $y_{i,t,j} = 1$ detected in the upper basin
- $y_{i,t,j} = 2$ detected in the lower basin
- $y_{i,t,j} = 3$ not detected

We use parameter-expanded data augmentation to account for the fact that the total number of adults in the population is unknown (Royle & Dorazio, 2012). Across the entire time period of the study, we assume N_s unique individuals have been alive in either basin. We observe N unique individuals across all surveys, where $N \leq N_s$. An estimate of N_s can be acquired by augmenting the observed capture histories with additional capture histories that consist entirely of non-detections, thus modeling a large number $M > N_s$ of individuals, $M - N_s$ of which never existed (Royle, 2009). Here, M was chosen to be 2182 (1212 observed unique individuals plus 970 augmented individuals). We verified that posterior estimates of N_s were much less than M to avoid problems on the boundary of this augmented parameter space (Dennis, Morgan & Ridout, 2015).

We denote the true state of individual i in primary period t as $s_{i,t}$. We assume that within a primary period, the state of each individual does not change. This assumption is justified by the short time intervals between secondary periods within primary periods. Four states are possible:

- $s_{i,t} = 1$ alive in the upper basin
- $s_{i,t} = 2$ alive in the lower basin
- $s_{i,t} = 3$ not recruited
- $s_{i,t} = 4$ dead

The "not recruited" state applies to individuals that have not yet entered the population, including individuals that haven't reached adulthood or pseudo-individuals created via data augmentation (as described above).

Observation model. An emission matrix $\mathbf{\Omega}^{(t)}$ links observations to hidden states for primary period t. The rows in $\mathbf{\Omega}^{(t)}$ correspond to the state of an individual in primary period t, and the columns correspond to observation probabilities such that the entry in the mth row, n^{th} column is $Pr(y_{i,t,j} = n \mid s_{i,t} = m)$:

$$\mathbf{\Omega}^{(t)} = \begin{array}{c} \\ \end{array} \begin{matrix} \text{Detected: upper} & \text{Detected: lower} & \text{Not detected} & \\ \end{matrix}$$

$$\mathbf{\Omega}^{(t)} = \begin{pmatrix} p_t & 0 & 1 - p_t \\ 0 & p_t & 1 - p_t \\ 0 & 0 & 1 \\ 0 & 0 & 1 \end{pmatrix} \begin{matrix} \text{Alive: upper} \\ \text{Alive: lower} \\ \text{Not recruited} \\ \text{Dead} \end{matrix}$$

(columns: Detected: upper, Detected: lower, Not detected)

Here, p_t is the probability of detection for an individual if it is alive in primary period t. We allowed detection probabilities to vary over time (Joseph & Knapp, 2018):

$$\text{logit}(p_t) = \alpha_0 + \epsilon_t^{(p)},$$

where α_0 is an intercept parameter, and $\epsilon_t^{(p)}$ is an adjustment on detection probability for primary period t.

State model. The hidden states of each individual evolve as a Markov process with transition matrix $\mathbf{\Psi}^{(i,t)}$, where the element in the mth row, n^{th} column is $\Pr(s_{i,t+1} = n \mid s_{i,t} = m)$:

$$
\begin{array}{c}
\\
\mathbf{\Psi}^{(i,t)} =
\end{array}
\begin{array}{cccc}
\text{Alive: upper} & \text{Alive: lower} & \text{Not recruited} & \text{Dead} \\
\end{array}
$$

$$
\mathbf{\Psi}^{(i,t)} = \begin{pmatrix}
\phi_{i,t}(1 - \nu^{(l)}) & \phi_{i,t}\nu^{(l)} & 0 & 1 - \phi_{i,t} \\
\phi_{i,t}\nu^{(u)} & \phi_{i,t}(1 - \nu^{(u)}) & 0 & 1 - \phi_{i,t} \\
\gamma_t\rho^{(u)} & \gamma_t(1 - \rho^{(u)}) & 1 - \gamma_t & 0 \\
0 & 0 & 0 & 1
\end{pmatrix}
\begin{array}{c}
\text{Alive: upper} \\
\text{Alive: lower} \\
\text{Not recruited} \\
\text{Dead}
\end{array}
$$

Here, $\phi_{i,t}$ is the probability of survival, $\nu^{(l)}$ and $\nu^{(u)}$ are the probabilities of moving to the lower or upper basin respectively (conditional on survival), γ_t is the probability of recruitment, and $\rho^{(u)}$ is the probability of recruiting into the upper basin conditional on recruitment.

Survival probabilities were modeled as a function of Bd load:

$$\text{logit}(\phi_{i,t}) = \beta_0 + \beta^{(g)}_{\text{g[i]}} + \beta^{(z)}_{\text{g[i]}} z_{i,t},$$

where β_0 is an intercept parameter, $\beta^{(g)}_{\text{g[i]}}$ is an adjustment for group g, $g[i]$ is the group that individual i belongs to, $\beta^{(z)}_{\text{g[i]}}$ is the effect of Bd load on group g, and $z_{i,t}$ is the Bd load of individual i in primary period t.

We treat the field experiment in 2015 as the first primary period, for which states are known for experimental animals. For example, if individual i was captured and released at the upper basin, then we know that $s_{i,t} = 1$. Initial states are not known for non-experimental animals, which could have been alive in either basin (states 1 or 2), or in the not recruited class (state 3). Note that we are only interested in modeling the state and capture histories of animals that might have been alive. We assigned a Dirichlet(1, 1,

1) prior for the initial state distribution for non-experimental frogs, which assigns equal prior density to each initial state.

Bd load model. We modeled Bd loads as being normally distributed on a transformed scale. Raw Bd loads were transformed using a $\log_{10} + 1$ transformation, then centered and scaled to have mean zero and unit standard deviation (in an attempt to avoid ill-conditioning, as expected Bd load is used in the detection and survival model components). Let $z_{i,t}^{\text{obs}}$ represent the observed transformed Bd load, and $z_{i,t}$ represent the expected value for individual i on primary period t. The observation model for Bd loads of detected individuals was Gaussian on the transformed scale:

$$z_{i,t}^{\text{obs}} \sim \text{Normal}(z_{i,t}, \sigma),$$

where σ is an observation-level standard deviation parameter.

Expected Bd load was modeled as a function of treatment, primary period, and individual identity:

$$z_{i,t} = \mu_t + \beta_{\text{trt}[i]} + \epsilon_i,$$

where μ_t is a time-varying intercept, $\beta_{\text{trt}[i]}$ is an adjustment for the treatment group of individual i (denoted $\text{trt}[i]$) to account for differences in mean Bd loads between treated, control, and non-experimental animals, and ϵ_i is an individual-level adjustment.

Prior distributions. We expected movement among basins to be rare, so both movement parameters ($\nu^{(l)}$ and $\nu^{(u)}$) were assigned Beta(2, 20) priors. Primary period and individual-level adjustments were modeled using zero-mean normal distributions with unknown standard deviations specific to the process of interest, e.g., for the probability of entering the population: $[\lambda_{1:T}] = \prod_{t=1}^{T} \text{Normal}(\lambda_t | 0, \sigma^{(\lambda)})$. Standard deviation parameters were given unit scale half normal priors, and all remaining parameters were given unit

scale normal priors.

Inference. We sampled from the posterior distribution of this model using dynamic Hamiltonian Monte Carlo in Stan. We drew 3000 iterations for each of four chains, using a maximum treedepth of 11 and an adapt_delta value of 0.99. Convergence was checked by visual inspection of traceplots and with Rhat values, using Rhat $<$ 1.1 as a threshold. Models were fit using the rstan R package, version 2.21.2 (Stan Development Team, 2020).

Results

Based on CMR modeling, across the entire duration of the experiment (2015-2018), the 1206 unique individuals included in the study were estimated to represent approximately 80% (posterior median) of the adults that existed in the LeConte population during this time (CI: 75% – 88%). Between frog release in 2015 and the final survey in 2018, seven recaptured individuals moved between the two basins. All seven were in the treated group and moved from the upper to the lower basin. These individuals were included in counts of unique individuals in the basin in which they were captured. In CMR surveys conducted during 2016-2018, a total of 2208 adult frogs were captured, representing 831 unique individuals. Of the 745 unique frogs captured in the lower basin, 132 were in the treated group, two were in the control group, and 611 were not part of the original treatment experiment ("non-experimental" frogs). In the upper basin, 89 unique frogs were captured, of which 81 were in the treated group and eight were non-experimental. No control frogs were captured in the upper basin.

Frog detection probabilities in the study populations varied over time, but overall were comparable to estimates previously reported from other populations (Joseph & Knapp, 2018). The primary period with the highest detection probabilities had a posterior median detection probability of 0.52 (CI: 0.49, 0.56). In contrast, the primary period with the lowest detection probabilities had a posterior median of 0.1 (CI: 0.05, 0.17). On an average

primary period, posterior median detection probability was 0.28 (CI: 0.17, 0.44).

Itraconazole treatment of adults – Treasure Lakes Basin

Methods

In 2018, we detected a Bd epizootic in the only Bd-naive *R. sierrae* population remaining in the Treasure Lakes Basin (Table S1), providing another opportunity to test the effectiveness of antifungal treatment on adult frogs. The treatment was conducted as a management action instead of an experiment because relatively few adults remained in this population at the time of the treatment. As such, dividing the frogs into treated and control groups would have provided little statistical power to detect between-group differences. Although the lack of an experimental design limits the generality of our findings, the treatment is included here because of the additional insights the results provide. Specifically, the greater range of treatment days to which frogs were exposed (compared to the LeConte treatments) provided an opportunity to evaluate the effectiveness of itraconazole treatment on Bd loads as a function of the number of daily treatments frogs received.

We used the same methods as described for the LeConte treatments, with two important differences: (1) all frogs were treated (there was no control group), and (2) new frogs were captured from the lake and added to the pens during the first five days of the 7-day treatment period. We captured and treated 28 frogs on 16 July 2018, then added and treated an additional 24, 7, 7, 4, and 4 frogs on July 17 through 21, respectively. Although we captured and treated a total of 74 frogs, we released only 33 live frogs at the end of the treatment due to chytridiomycosis-caused mortality throughout the treatment period (mortality rate = 55%). In addition to swabs collected from all frogs immediately following their initial capture, we also collected swabs from each surviving frog after the final itraconazole treatment. To describe post-treatment frog-Bd dynamics, we conducted

VES and CMR surveys one month after the 2018 treatment (August 21-23), and again in 2019 (August 15-16) and 2020 (June 23-25).

We assessed treatment effectiveness by comparing Bd loads measured before and after treatment, using the model *bd_load ~ trt_period* (family = negative binomial, trt_period = [begin, end]). To evaluate the effectiveness of itraconazole treatment on Bd loads as a function of the number of daily treatments frogs received, we calculated treatment effectiveness for individual frogs as the negative log ratio of pre-treatment to post-treatment Bd loads (hereafter, "LRR"): $-\log_{10}((\text{load}_{\text{pre}} + 1)/(\text{load}_{\text{post}} + 1)$. Larger absolute values of LRR indicate a larger reduction in Bd load. To evaluate the factors influencing treatment effectiveness on individual frogs, we used the model *LRR ~ (capture_bdload_std x days_inside)* (family = gaussian, capture_bdload_std = Bd load prior to treatment standardized to mean = 0 and standard deviation = 1, days_inside = number of treatments a frog received).

Results

Similar to the situation in LeConte Basin, in the Treasure Lake study population Bd loads on adult frogs were very high prior to itraconazole treatment. Itraconazole treatment reduced Bd loads by more than two orders of magnitude, and model results affirmed this effect (Table S9). The number of itraconazole treatments a frog received ("days_inside") increased treatment effectiveness (Table S10). Initial Bd load ("capture_bdload_std") and the (days_inside x capture_bdload_std) interaction term were both unimportant (Table S10).

Of the 33 frogs that were released back into the lake following treatment, 16 were recaptured in the CMR survey conducted one month later (Figure S3). In addition, one non-experimental adult frog was captured, and one dead tagged (i.e., treated) adult was found. Bd loads of most recaptured frogs were low compared to those of frogs at the start

and end of the treatment period (Figure S3). There was no obvious relationship between the number of treatments a frog received and whether or not it was recaptured one month later (Figure S3). In surveys conducted in 2019 (the year following treatment) and 2020, we observed no *R. sierrae* of any life stage. Therefore, despite the substantial reduction in Bd loads caused by the 2018 treatment and the relatively large fraction of treated frogs recaptured one month later, few or no frogs survived overwinter until summer 2019.

Literature Cited

Dennis EB, Morgan BJ, Ridout MS. 2015. Computational aspects of n-mixture models. *Biometrics* 71:237–246.

Garner TWJ, Garcia G, Carroll B, Fisher MC. 2009. Using itraconazole to clear *Batrachochytrium dendrobatidis* infection, and subsequent depigmentation of *Alytes muletensis* tadpoles. *Diseases of Aquatic Organisms* 83:257–260.

Gelman A, Carlin JB, Stern HS, Dunson DB, Vehtari A, Rubin DB. 2013. *Bayesian data analysis*. Boca Raton, Florida, USA: CRC Press.

Harris RN, Brucker RM, Walke JB, Becker MH, Schwantes CR, Flaherty DC, Lam BA, Woodhams DC, Briggs CJ, Vredenburg VT, Minbiole KPC. 2009. Skin microbes on frogs prevent morbidity and mortality caused by a lethal skin fungus. *The ISME journal* 3:818–824.

Jani AJ, Knapp RA, Briggs CJ. 2017. Epidemic and endemic pathogen dynamics correspond to distinct host population microbiomes at a landscape scale. *Proceedings of the Royal Society B: Biological Sciences* 284:20170944.

Joseph MB, Knapp RA. 2018. Disease and climate effects on individuals drive post-reintroduction population dynamics of an endangered amphibian. *Ecosphere* 9:e02499.

Knapp RA, Fellers GM, Kleeman PM, Miller DA, Vredenburg VT, Rosenblum EB, Briggs CJ. 2016. Large-scale recovery of an endangered amphibian despite ongoing exposure to multiple stressors. *Proceedings of the National Academy of Sciences USA* 113:11889–11894.

Knapp RA, Matthews KR. 2000. Non-native fish introductions and the decline of the mountain yellow-legged frog from within protected areas. *Conservation Biology* 14:428–438.

Mosher BA, Huyvaert KP, Bailey LL. 2018. Beyond the swab: Ecosystem sampling to understand the persistence of an amphibian pathogen. *Oecologia* 188:319–330.

Rachowicz LJ, Briggs CJ. 2007. Quantifying the disease transmission function: Effects of density on *Batrachochytrium dendrobatidis* transmission in the mountain yellow-legged frog *Rana muscosa*. *Journal of Animal Ecology* 76:711–721.

Royle JA. 2009. Analysis of capture–recapture models with individual covariates using data augmentation. *Biometrics* 65:267–274.

Royle JA, Dorazio RM. 2012. Parameter-expanded data augmentation for Bayesian analysis of capture–recapture models. *Journal of Ornithology* 152:521–537.

Stan Development Team. 2020. RStan: The R interface to Stan.

Vehtari A, Gelman A, Gabry J. 2017. Practical Bayesian model evaluation using leave-one-out cross-validation and WAIC. *Statistics and Computing* 27:1413–1432.

Vredenburg VT, Knapp RA, Tunstall TS, Briggs CJ. 2010. Dynamics of an emerging disease drive large-scale amphibian population extinctions. *Proceedings of the National Academy of Sciences USA* 107:9689–9694.

Tables

Table S1:

Characteristics of all sites used in the antifungal treatment experiments.

Basin	Site ID	Experiment	Life stage	Category	Elevation (m)	Depth (m)	Area (m^2)
Barrett	11469	itraconazole	early	treated	3383	2.7	3875
Barrett	11491	itraconazole	early	treated	3530	5.0	2316
Barrett	11493	itraconazole	early	treated	3459	0.6	269
Barrett	11470	itraconazole	early	control	3383	5.0	3998
Barrett	10222	itraconazole	early	control	3554	5.2	10568
Barrett	11495	itraconazole	early	control	3459	1.0	970
Dusy	11518	itraconazole	early	treated	3408	1.0	2002
Dusy	11526	itraconazole	early	treated	3219	1.9	2966
Dusy	11506	itraconazole	early	treated	3469	1.8	1414
Dusy	11517	itraconazole	early	control	3395	0.8	816
Dusy	11525	itraconazole	early	control	3158	1.2	2604
LeConte	10101	itraconazole	adult	treated	3213	1.5	5187
LeConte	10100	itraconazole	adult	treated	3298	14.9	25974
Treasure	50839	itraconazole	adult	treated	3410	11.0	34317
Dusy	11518	itracon + Jliv	subadult	treated	3408	1.0	2002

Life stage = "early" indicates tadpoles and subadults

Treatment = "itracon + Jliv" indicates the use of itraconazole and *J. lividum*

Table S2:

Timelines for antifungal treatment experiments conducted in the lower and upper basins of the LeConte study area in 2015.

(a) Lower basin

**Day 1 (24-Aug)**	**Day 2 (25-Aug)**	**Day 3 (26-Aug)**	**Day 4 (27-Aug)**	**Day 5-7 (28-30 Aug)**	**Day 8 (31-Aug)**	**Day 9 (1-Sep)**
Captured 50 frogs for "treated" group, Day 1 frogs swabbed and treated.	Captured 157 frogs for "treated" group, Day 2 frogs swabbed, Day 1 & 2 frogs treated.	Captured 152 frogs for "treated" group, Day 3 frogs swabbed, Day 1-3 frogs treated.	Captured 102 frogs for "control" group, swabbed and released. Day 1-3 frogs treated.	Day 1-3 frogs treated.	Day 1-3 frogs treated. Swabbed subset of Day 1, 2, and 3 frogs.	Day 1-3 frogs treated, released back into lakes.

(b) Upper basin

**Day 1 (8-Sep)**	**Day 2 (9-Sep)**	**Day 3 (10-Sep)**	**Day 4-6 (11-13 Sep)**	**Day 7 (14-Sep)**	**Day 8 (15-Sep)**
Captured 45 frogs for "treated" group, Day 1 frogs swabbed and treated.	Captured 161 frogs for "treated" group, Day 2 frogs swabbed, Day 1 & 2 frogs treated.	Captured 74 frogs for "control" group, swabbed and released, Day 1 & 2 frogs treated.	Day 1 & 2 frogs treated.	Day 1 & 2 frogs treated. Swabbed subset of Day 1 & 2 frogs.	Day 1 & 2 frogs treated, released back into lakes.

Table S3:

For the itraconazole treatment experiments in Barrett and Dusy basins, results of model comparing Bd loads on frogs in ponds assigned to control and treated groups immediately before the treatment period.

Model family is negative binomial.

	Estimate	Est. Error	lo95%CI	up95%CI	Rhat	Bulk ESS	Tail Ess
**Population-level effects**							
Intercept	11.99	0.55	11.05	13.21	1.00	2273	1948
treatment(treated)	0.15	0.77	-1.39	1.64	1.00	1956	1993
basin(dusy)	-1.36	0.70	-2.81	-0.02	1.00	2058	1797
treatment(treated):basin(dusy)	1.36	0.93	-0.54	3.17	1.00	1842	1705
**Family-specific parameters**							
overdispersion	0.30	0.04	0.23	0.39	1.00	2777	2617

Table S4:

For the itraconazole treatment experiments in Barrett and Dusy basins, results of model comparing Bd loads on frogs assigned to the treated group from before versus the end of the treatment period.

Model family is negative binomial.

	Estimate	Est. Error	lo95%CI	up95%CI	Rhat	Bulk ESS	Tail Ess
**Population-level effects**							
Intercept	13.46	0.27	12.96	14.00	1.00	3780	3096
overdispersion-Intercept	-1.59	0.12	-1.84	-1.35	1.00	4561	3194
stage(tadpole)	-2.28	0.29	-2.87	-1.74	1.00	3248	3010
trt_period(end)	-2.56	0.22	-2.98	-2.11	1.00	3179	2841
basin(dusy)	1.71	0.19	1.35	2.06	1.00	3680	3357
trt_period(end):basin(dusy)	-4.82	0.45	-5.68	-3.88	1.00	3125	2537
overdispersion-stage(tadpole)	1.36	0.14	1.10	1.64	1.00	4018	2923
overdispersion-trt_period(end)	-1.11	0.11	-1.34	-0.90	1.00	3478	3082
overdispersion-basin(dusy)	-0.83	0.12	-1.05	-0.59	1.00	4047	2579

Table S5:

For the Dusy Basin itraconazole treatment experiment, results of model comparing zoospore pools of ponds assigned to the control and treated groups before and after treatment.

Model family is negative binomial.

	Estimate	Est. Error	lo95%CI	up95%CI	Rhat	Bulk ESS	Tail Ess
**Group-level effects**							
sd(Intercept)	1.98	0.28	1.46	2.55	1.01	471	618
**Population-level effects**							
Intercept	8.65	0.64	7.42	9.89	1.00	558	987
pre_post(post)	1.74	0.94	-0.13	3.54	1.01	467	727
tmt(treated)	1.19	1.02	-0.87	3.20	1.01	627	886
pre_post(post):tmt(treated)	-2.54	1.28	-5.07	0.00	1.01	525	947
**Family-specific parameters**							
overdispersion	3.04	0.64	1.87	4.42	1.00	787	703

Table S6:

For the LeConte Basin itraconazole treatment experiment, results of model comparing Bd loads on frogs assigned to the control and treated categories immediately before the treatment period.

Model family is negative binomial.

	Estimate	Est. Error	lo95%CI	up95%CI	Rhat	Bulk ESS	Tail Ess
**Population-level effects**							
Intercept	17.01	0.13	16.75	17.27	1.00	2262	2396
location(upper)	0.03	0.20	-0.36	0.44	1.00	1663	2309
group(treated)	-0.90	0.16	-1.21	-0.60	1.00	2185	2317
location(upper):group(treated)	0.51	0.25	0.00	1.01	1.00	1679	2222
**Family-specific parameters**							
overdispersion	0.54	0.03	0.49	0.59	1.00	3333	2393

Table S7:

For the LeConte Basin itraconazole treatment experiment, results of model comparing Bd loads on frogs assigned to the treated group from before versus the end of the treatment period.

Model family is negative binomial.

	Estimate	Est. Error	lo95%CI	up95%CI	Rhat	Bulk ESS	Tail Ess
**Population-level effects**							
Intercept	16.11	0.11	15.90	16.32	1.00	4204	3258
location(upper)	0.54	0.19	0.16	0.93	1.00	3486	3216
trt_period(endtreat)	-2.66	0.22	-3.07	-2.23	1.00	3170	3162
location(upper):trt_period(endtreat)	1.15	0.38	0.41	1.89	1.00	3040	2980
**Family-specific parameters**							
overdispersion	0.31	0.01	0.29	0.34	1.00	3988	2862

Table S8:

For the LeConte Basin itraconazole treatment experiment, results of model comparing Bd loads on frogs in the treated group that survived versus died.
Model family is bernoulli.

	Estimate	Est. Error	lo95%CI	up95%CI	Rhat	Bulk ESS	Tail Ess
**Population-level effects**							
Intercept	-1.90	0.90	-3.70	-0.20	1.00	2120	2090
lbd_load	0.09	0.14	-0.18	0.36	1.00	2050	2081
location(upper)	0.11	2.02	-3.85	4.15	1.00	1460	1663
lbd_load:location(upper)	0.11	0.29	-0.48	0.68	1.00	1434	1693

Table S9:

For the Treasure Lakes Basin itraconazole treatment, results of model comparing Bd loads on frogs before versus the end of the treatment period.
Model family is negative binomial.

	Estimate	Est. Error	lo95%CI	up95%CI	Rhat	Bulk ESS	Tail Ess
**Population-level effects**							
Intercept	15.45	0.22	15.05	15.91	1.00	3452	2500
trt_period(after)	-1.35	0.41	-2.10	-0.50	1.00	3817	2448
**Family-specific parameters**							
overdispersion	0.29	0.03	0.23	0.35	1.00	3580	2946

Table S10:

For the Treasure Lakes Basin itraconazole treatment, results of model evaluating predictors of treatment effectiveness.

Model family is gaussian.

	Estimate	Est. Error	lo95%CI	up95%CI	Rhat	Bulk ESS	Tail Ess
**Population-level effects**							
Intercept	3.13	2.68	-2.15	8.40	1.00	1800	2078
capture_bdload_std	-5.93	4.88	-15.25	4.22	1.00	1357	1773
days_inside	-1.42	0.43	-2.26	-0.57	1.00	1820	2119
capture_bdload_std:days_inside	0.56	0.75	-0.99	1.99	1.00	1351	1549
**Family-specific parameters**							
sigma	3.10	0.43	2.40	4.07	1.00	2484	2221

Table S11:

For the Dusy Basin microbiome augmentation experiment, results of model comparing Bd loads on frogs assigned to the control and treated groups immediately before the itraconazole treatment period.

Model family is negative binomial.

	Estimate	Est. Error	lo95%CI	up95%CI	Rhat	Bulk ESS	Tail Ess
**Population-level effects**							
Intercept	13.97	0.22	13.57	14.42	1.00	3514	2409
expt_trt(treated)	0.17	0.27	-0.37	0.68	1.00	3111	2460
**Family-specific parameters**							
overdispersion	0.82	0.12	0.61	1.07	1.00	3564	2992

Table S12:

For the Dusy Basin microbiome augmentation experiment, results of model comparing Bd loads on frogs assigned to the treated category from immediately before versus at the end of the itraconazole treatment.

Model family is zero-inflated negative binomial.

	Estimate	Est. Error	lo95%CI	up95%CI	Rhat	Bulk ESS	Tail Ess
**Population-level effects**							
Intercept	14.15	0.13	13.90	14.42	1.00	5206	2934
days(0)	-8.92	0.22	-9.35	-8.47	1.00	3798	2834
**Family-specific parameters**							
overdispersion	1.10	0.16	0.79	1.43	1.00	3980	2962
zi	0.21	0.04	0.13	0.29	1.00	3980	2480

Figures

Figure S1

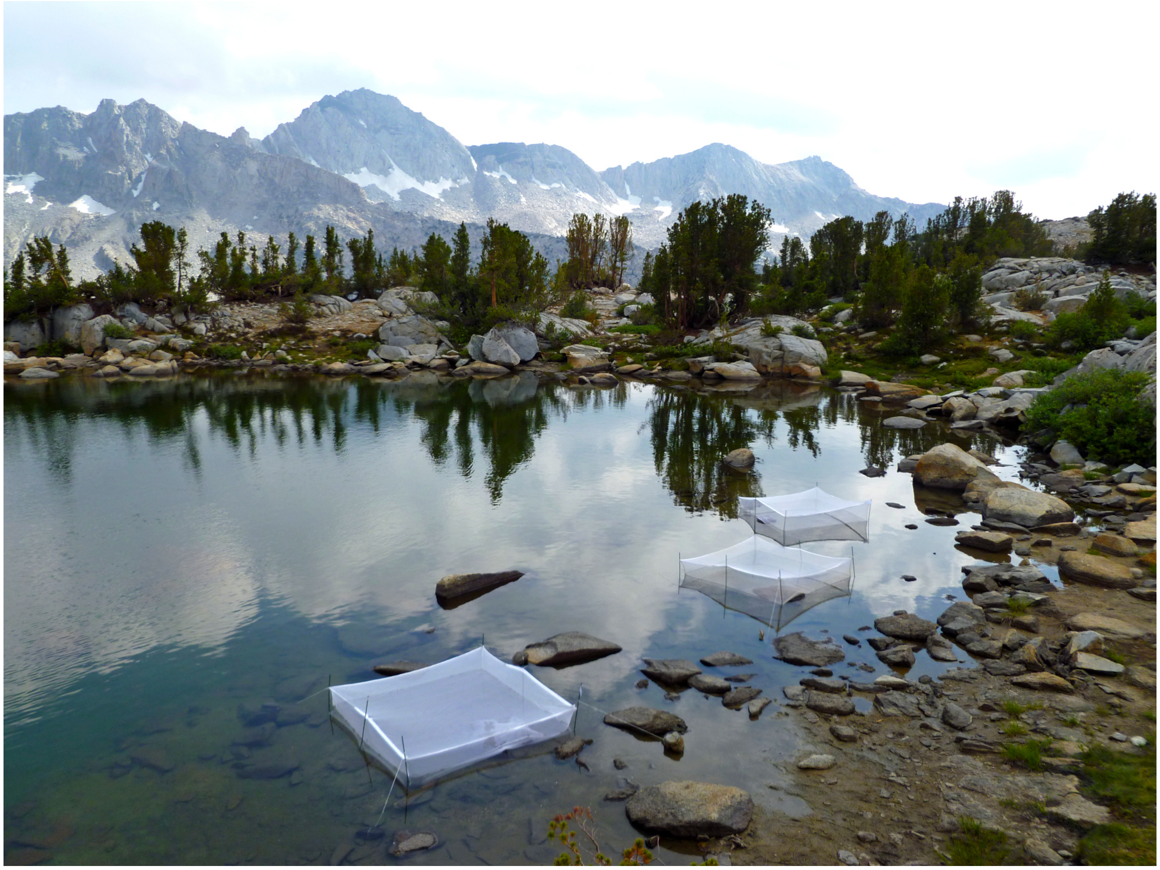

Photograph showing mesh pens used to hold subadult *R. sierrae* during the 2012 microbiome augmentation experiment in Dusy Basin.

Photo credit: Roland Knapp

Figure S2

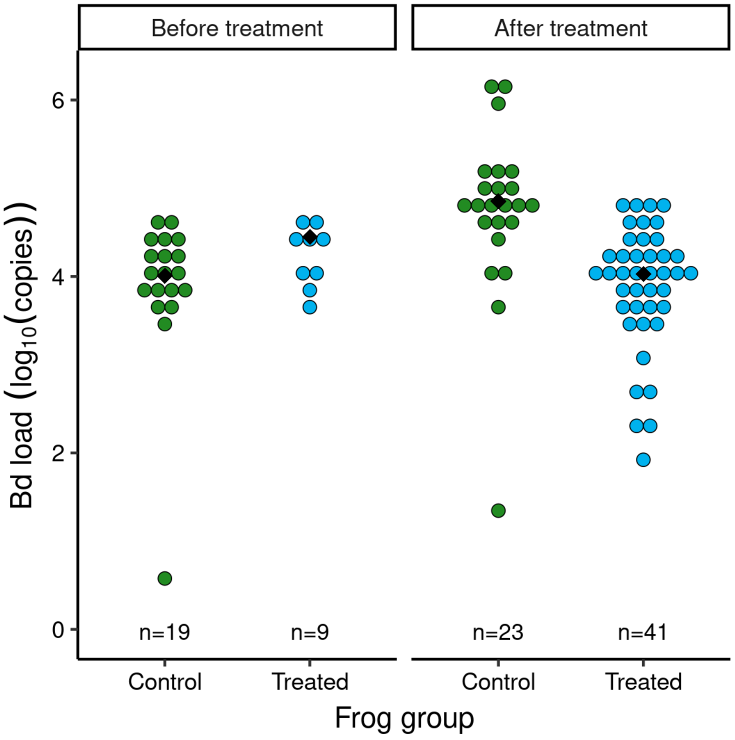

In the Dusy Basin study ponds assigned to the control or treated groups, zoospore pools measured before and after itraconazole treatment of *R. sierrae*. The y-axis displays Bd load per water sample, normalized to a 1-liter sample volume. Each dot represents a single sample, and median values for each treatment period are indicated with a black diamond. The number of samples included is displayed above the x-axis.

Figure S3

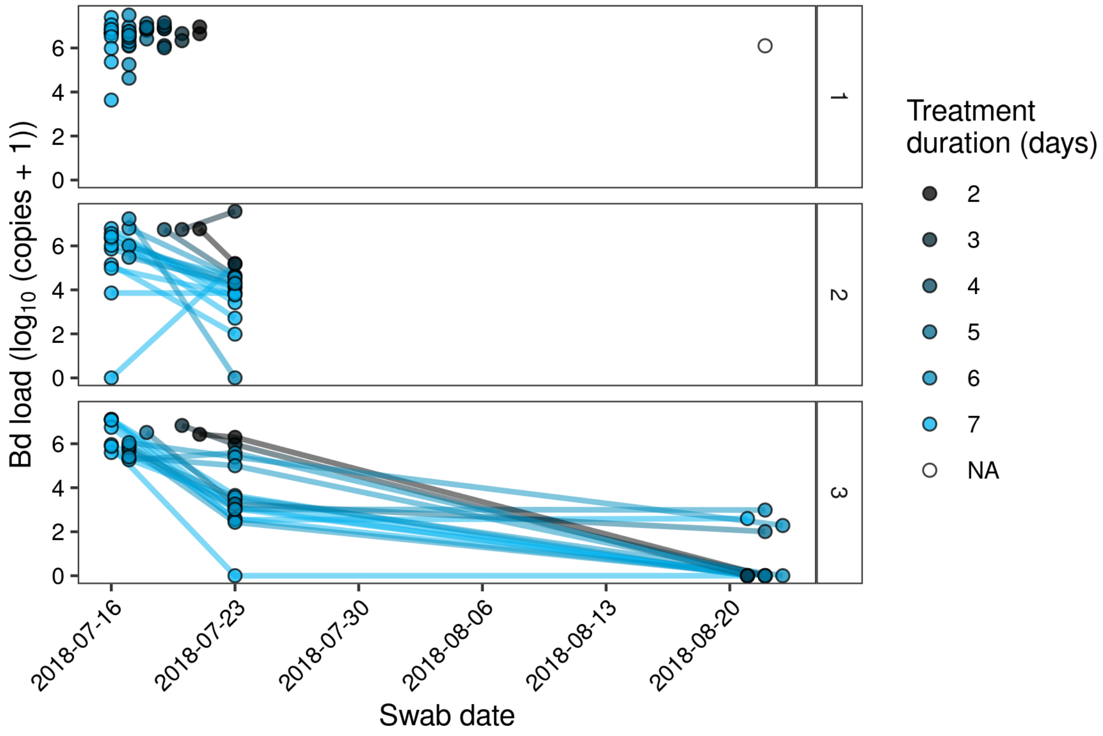

For all adult *R. sierrae* in the 2018 Treasure Lakes itraconazole treatment, Bd loads over a two month period that includes the July treatment (16 July to 23 July 2018) and August follow-up surveys.

Points from the same frog are connected by a line. Panel labels are as follows: 1 = frog that died during the treatment period, 2 = survivor that was not recaptured during the post-release survey in August, and 3 = survivor that was recaptured during the August post-release survey. The single non-experimental frog captured in August was not included in the treatment (treatment duration = "NA") and is included in panel 1.

Figure S4

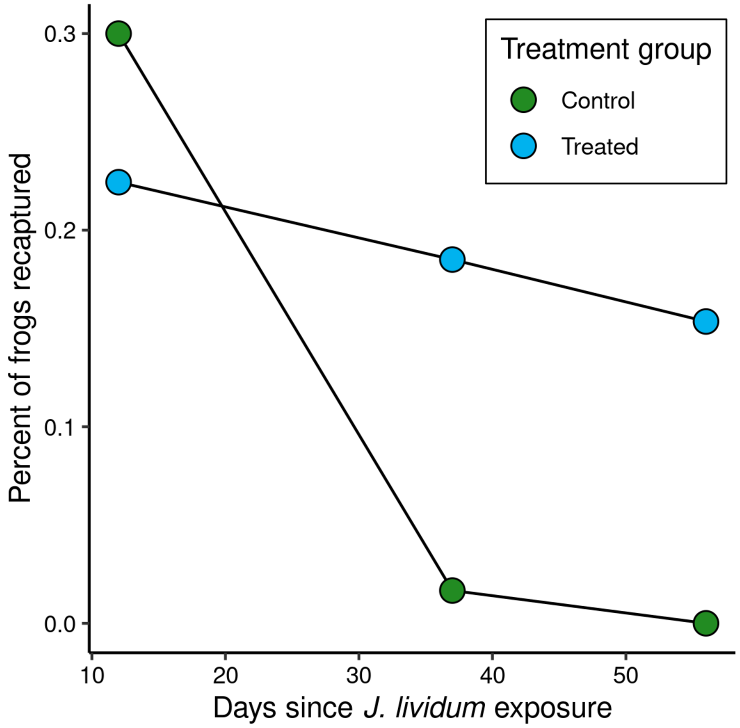

In the Dusy Basin *J. lividum* augmentation experiment, the percent of frogs in the treated and control groups recaptured during the two months following *J. lividum* exposure.

The number of subadults captured during each survey is given in Figure 6.
